# Establishment of a transparent soil system to study *Bacillus subtilis* chemical ecology

**DOI:** 10.1038/s43705-023-00318-5

**Published:** 2023-10-14

**Authors:** Carlos N. Lozano-Andrade, Carla G. Nogueira, Nathalie N. S. E. Henriksen, Mario Wibowo, Scott A. Jarmusch, Ákos T. Kovács

**Affiliations:** 1 DTU Bioengineering, Technical University of Denmark, 2800 Kgs Lyngby, Denmark; 2 Institute of Biology, Leiden University, 2333 BE Leiden, The Netherlands

**Keywords:** Soil microbiology, Bacteriology, Microbial ecology

## Abstract

Bacterial secondary metabolites are structurally diverse molecules that drive microbial interaction by altering growth, cell differentiation, and signaling. *Bacillus subtilis*, a Gram-positive soil-dwelling bacterium, produces a wealth of secondary metabolites, among them, lipopeptides have been vastly studied by their antimicrobial, antitumor, and surfactant activities. However, the natural functions of secondary metabolites in the lifestyles of the producing organism remain less explored under natural conditions, i.e. in soil. Here, we describe a hydrogel-based transparent soil system to investigate *B. subtilis* chemical ecology under controllable soil-like conditions. The transparent soil matrix allows the growth of *B. subtilis* and other isolates gnotobiotically and under nutrient-controlled conditions. Additionally, we show that transparent soil allows the detection of lipopeptides production and dynamics by HPLC-MS, and MALDI-MS imaging, along with fluorescence imaging of 3-dimensional bacterial assemblages. We anticipate that this affordable and highly controllable system will promote bacterial chemical ecology research and help to elucidate microbial interactions driven by secondary metabolites.

## Introduction

Soil microbial communities play a pivotal role in biogeochemical cycles and ecosystem stability on a global scale [1–3]. These communities encompass thousands of interacting species that mediate essential ecosystem services, ranging from biomass decomposition to crop productivity [4, 5]. It is understood that in the spatially and temporally heterogenous environment of soil, microbes produce a plethora of specialized or secondary metabolites (SM) that can function as key mediators of microbial interactions. These metabolites allow microbes to engage in complex behaviors, such as cooperation, competition, and communication, which are important for shaping the composition and functionality of soil microbial communities [6]. Despite extensive efforts to unravel how secondary metabolites impact microbiomes and soil processes, significant challenges persist in elucidating their precise roles in the natural environment. One of the main constraints is the difficulty in detecting and quantifying SMs in soil due to the matrix’s complexity in terms of microbial diversity, physical properties, and chemical composition. This limitation hinders our ability to track and determine the fate of SMs, which impedes our understanding of the underlying mechanisms of microbial processes mediated by these small molecules and their impact on ecosystem-level functions [7, 8].

To address these challenges, previous studies have employed soil-like or transparent soil (TS) matrices to establish controlled systems for studying soil-microbe interactions. These matrices provide an accessible substrate for imaging and chemical analysis that facilitate our understanding of biological processes. They are designed as an intermediate alternative, being more complex than liquid media but simpler than natural soils, facilitating well-replicated studies with higher statistical power [9–14]. Hitherto, phytoagar, peat, mineral substrates (such as calcined clay), and hydroponic systems have all been successfully used as reductionist approaches to examine soil-plant-microbe interactions [9, 13–18]. Similarly, TS, originally developed for applications in hydrology and geology, has been adapted as a soil-mimicking model to overcome the opaqueness of natural soil, easing the study of biological processes in real-time by coupling imaging technologies. For instance, Downie et al. used a TS composed of Nafion particles, a polymer with a low refractive index, to explore bacterial colonization and distribution patterns on plant roots [10, 11]. Likewise, Sharma et al. assessed Nafion particles embedded in a microfluidic chamber as microcosms for cell imaging and detection of microbial activity at the single-cell level using Raman microspectroscopy [12].

All these examples have significantly contributed toward elucidating the underlying mechanisms of microbial processes which are otherwise difficult to study in natural soil. However, the dynamics of secondary metabolites (SMs) are not investigated by these approaches and SM driven interactions are not dismantled under soil-mimicking conditions. Therefore, we used a hydrogel-based transparent soil developed by Ma et al., initially intended for in vivo plant root phenotyping, as a gnotobiotic system to explore SM production and bacterial population dynamics in an intermediate setting between planktonic culture and natural soils [13].

As a proof of concept, we demonstrated the feasibility of this hydrogel matrix system for studying the growth and lipopeptide production of a wild isolate from the *B. subtilis* complex. Strains belonging to this group have been widely proposed as pivotal soil-dwelling bacteria, positively affecting plant development and soil health through multiple mechanisms, including secondary metabolite production [19–22]. We present a detailed protocol for preparing and using the hydrogel matrix in conjunction with microbiological and analytical chemistry techniques, such as flow cytometry, LC-MS, and MALDI MS imaging, to track bacterial growth and secondary metabolite production over time.

Moreover, we evaluated the versatility and applicability of this system to a set of bacterial species by demonstrating the ability of other bacterial isolates to grow in the hydrogel matrix system. Finally, we grew tomato plants in the hydrogel matrix system and evaluated the ability of *B. subtilis* to colonizes the tomato roots, confirming the potential of this system for studying plant-microbe interactions.

## Materials and methods

### Bacterial strains

All the bacterial strains used in this study are shown in Table 1. *B. subtilis* strains were routinely maintained in lysogeny broth (LB) medium (LB-Lennox, Carl Roth; 10 g/l tryptone, 5 g/l yeast extract, and 5 g/l NaCl) at 37 °C with shaking at 220 rpm (1.9 cm orbit), while other isolates were grown in using 0.1 × TSB (tryptic soy broth, CASO Broth, Sigma-Aldrich) under similar incubation parameters.

**Table 1:** Detailed information about strains used in this study.

Strain	Description	Reference
P5_B1	*B. subtilis* soil isolate from sampling site 55.788800, 12.558300	[23, 58]
DTUB38	P5_B1 *amyE*::P_hyperspank_-*gfp* (Chl^R^)	[69]
DTUB148	P5_B1 *amyE*::P_hyperspank_-*gfp* (Chl^R^); *srfAC*::Tn*10* (Spec^R^)	
D749	*Pedobacter* sp. soil isolate from sampling site 55.788800, 12.558300	[70]
D757	*Rhodococcus globerulus s*oil isolate from sampling site 55.788800, 12.558300	
D763	*Stenothrophomonas indicatrix* soil isolate from sampling site 55.788800, 12.558300	
D764	*Chryseobacterium* sp. soil isolate from sampling site 55.788800, 12.558300	

### Hydrogel transparent soil production

The microcosm experiments were conducted using the hydrogel transparent soil previously described by Ma et al. [13]. Briefly, autoclaved polymeric solution containing 2.4 g/l sodium alginate and 9.6 g/l Phytagel (Sigma-Aldrich) was dropped into a stirred solution of CaCl_2_ 2%, allowing for the rapid formation of spherical beads. Subsequently, the beads were soaked in 0.1 × TSB as a nutrient solution for 2 h. Later, the excess liquid was drained, and 25 g of beads were transferred into 50 ml Falcon tubes or LEGO boxes as the experimental units for all subsequent experiments (Fig. S1). All the solutions and materials were autoclaved to ensure sterility of the system. The microcosms were prepared right before conducting each experiment; however, the hydrogel beads can be storage a 4 °C with any apparent changes.

### Bacterial population dynamics on transparent soil microcosm

As the hydrogel matrix was originally designed for plant growth, the aim of our first experiment was to evaluate its potential as a growth substrate for bacterial cultures when the beads were supplemented with 0.1 × TSB. We evaluated the population dynamics of *B. subtilis* P5_B1 and set of bacterial isolates, either individually in monoculture or as mixed bacterial assemblage, using a variety of techniques including colony counting, flow cytometry, and culture-independent 16S rRNA on the hydrogel microcosms.

For the monoculture assay, the bacterial strains were initially diluted to 1 × 10^6^ CFU/ml in 0.1 × TSB. Subsequently, 2.5 ml of each culture was inoculated into hydrogel matrix microcosms (25 g in a 50 ml Falcon). These microcosms were kept at 21 °C under static conditions. At days 1, 3, 5, 8, 11, and 15, one gram of beads was transferred into a 15 ml Falcon tube, diluted in 0.9% NaCl, and vortexed for 10 min. To determine colony-forming units (CFU), 100 µl of each sample was serially diluted and spread onto 0.1 × TSA (tryptic soy agar, Sigma-Aldrich), and the CFUs were estimated after 3 days. For spore counting, the same dilutions were heat-treated at 80 °C for 15 min to inactivate vegetative cells before plating the samples on 0.1 × TSA.

Furthermore, the growth dynamics of *B. subtilis* (P5_B1gfp) were monitored by flow cytometry. To do this, the samples were passed through a Miracloth (Millipore) to remove bead debris and diluted 1000-fold in 0.9% NaCl. Subsequently, 200 µl of the solution was analyzed on a flow cytometer (MACSQuant® VYB, Miltenyi Biotec). Green-fluorescent cells were detected using the blue laser (488 nm) and filter B1 (525/50 nm). Non-inoculated beads and 0.1 × TSB were used as control to identify background autofluorescence. For each sample, single events were identified from the FSC-H vs. FSC-A plot and gated into the GFP vs. FSC-A plot, where GFP-positive cells were identified.

Lastly, the population dynamic of a bacterial assemblage composed by all five strains was followed using culture-independent bacterial 16S gene profiling. To do so, overnight cultures of the five strains were OD adjusted on 0.1 × TSB to 1.0 (P5_B1_*gfp*_) and 0.01 (D749, D757, D763, and D764) and mixed on equal volumes. Then, 2.5 ml of the mixture were inoculated into the transparent soil microcosms at incubated at 21 °C. At days 1, 3, 5, 8, 11 and 15, the bacterial assemblage genomic DNA was extracted from 1 g of beads using DNeasy PowerSoil Pro kit (QIAGEN) following the manufacturer’s instructions. The hypervariable regions V3-V4 of the 16S rRNA gene was PCR-amplified using Fw_V3V4 (5’-CCTACGGGNGGCWGCAG-3’) and Rv_V3V4 (5’-GACTACHVGGGTATCTAATCC-3’) primers tagged with eight nucleotides length barcodes as reported by Kiesewalter et al. [23]. The PCR reactions contained 10.6 μl DNase-free water, 12.5 μl TEMPase Hot Start 2x Master Mix, 0.8 μl of each primer (10 μM), and 0.3 μl of 50 ng/µl DNA template. The PCR was performed using the conditions of 95 °C for 15 min, followed by 30 cycles of 95 °C for 30 s, 62 °C for 30 s, 72 °C for 30 s, and finally, 72 °C for 5 min. All V3-V4 amplicons were purified using the NucleoSpin gel and PCR cleanup kit (Macherey-Nagel) and pooled in equimolar ratios. The amplicon pool was submitted to Novogene Europe Company Limited (United Kingdom) for high-throughput sequencing on an Illumina NovaSeq 6000 platform with 2 million reads (2 × 250 bp paired-end reads). The sequence data processing was conducted using the QIIME 2 pipeline [24]. The paired-end reads were demultiplexed (cutadapt), denoised and merged using cutadapt [25] and DADA2 [26], respectively. The 16S rRNA reference sequences with a 99% identity criterion obtained from the SILVA database release 132 were trimmed to the V3-V4 region, bound by the primer pair used for amplification, and the product length was limited to 200–500 nucleotides [27]. The taxonomy was assigned to the sequences in the feature table generated by DADA2 by using the VSEARCH-based consensus taxonomy classifier [28]. Relative species abundance, as population dynamic parameter, was estimated by importing the QIIME 2 artefacts into the R software (4.1) [29] with the package qiime2R and further processed using phyloseq [30] and dplyr [31]. All the graphical visualizations were made with ggplot2 [32]. At least three replicates were conducted for all experiments. The raw sequence data were deposited in NCBI SRA under the accession: PRJNA982715.

### Extraction and detection of secondary metabolites from transparent soil microcosms

To extract secondary metabolites from the hydrogel beads, 1 g of beads was mixed with 4 ml of isopropylalcohol:EtOAc (1:3, v/v) containing 1% formic acid followed by vortexing the tubes briefly. Next, the tubes were sonicated for 60 min. The organic solvent was transferred to a new tube, evaporated to dryness under N_2_, and re-dissolved in 300 µl of methanol for further sonication over 15 min. After centrifugation at 13,400 rpm for 3 min, the supernatants were transferred to HPLC vials and subjected to ultrahigh-performance liquid chromatography-high resolution mass spectrometry (UHPLC-HRMS) analysis.

UHPLC-HRMS was performed on an Agilent Infinity 1290 UHPLC system with a diode array detector. UV–visible spectra were recorded from 190 to 640 nm. Liquid chromatography of 1 or 5 µl extract (or standard solution) was performed using an Agilent Poroshell 120 phenyl-hexyl column (2.1 × 150 mm, 2.7 μm) at 60 °C using of acetonitrile (ACN) and H_2_O, both containing 20 mM formic acid, as mobile phases. Initially, a gradient of 10% ACN/H_2_O to 100% acetonitrile over 15 min was employed, followed by isocratic elution of 100% ACN for 2 min. The gradient was returned to 10% ACN/H_2_O in 0.1 min, and finally isocratic condition of 10% ACN/H_2_O for 2.9 min, at a flow rate of 0.35 ml/min. HRMS spectra were acquired in positive ionization mode on an Agilent 6545 QTOF MS equipped with an Agilent Dual Jet Stream electrospray ion source with a drying gas temperature of 250 °C, drying gas flow of 8 l/min, sheath gas temperature of 300 °C, and sheath gas flow of 12 l/min. Capillary voltage was set to 4000 V and nozzle voltage to 500 V. All solvents used for UHPLC-HRMS experiments were LC-MS grade (VWR Chemicals); while for metabolites extraction, the solvents were of HPLC grade (VWR Chemicals). MS data analysis and processing were performed using Agilent MassHunter Qualitative Analysis B.07.00.

A molecular network was created with the Feature-Based Molecular Networking (FBMN) workflow [33] on GNPS (https://gnps.ucsd.edu) [34] The mass spectrometry data were first processed with MZmine2 [35] and the results were exported to GNPS for FBMN analysis. The data was filtered by removing all MS/MS fragment ions within ±17 Da of the precursor *m*/*z*. MS/MS spectra were window filtered by choosing only the top 6 fragment ions in the ±50 Da window throughout the spectrum. The precursor ion mass tolerance was set to 0.005 Da and the MS/MS fragment ion tolerance to 0.025 Da. A molecular network was then created where edges were filtered to have a cosine score above 0.7 and more than 6 matched peaks. Further, edges between two nodes were kept in the network if and only if each of the nodes appeared in each other respective top 10 most similar nodes. Finally, the maximum size of a molecular family was set to 100, and the lowest scoring edges were removed from molecular families until the molecular family size was below this threshold. The spectra in the network were then searched against GNPS spectral libraries [34, 36]. The library spectra were filtered in the same manner as the input data. All matches kept between network spectra and library spectra were required to have a score above 0.7 and at least 6 matched peaks. The DEREPLICATOR was used to annotate MS/MS spectra [37]. Additional edges were provided by the user. The molecular networks were visualized using Cytoscape software [38]. The FBMN workflow can be found in GNPS and data is stored in MSV (https://gnps.ucsd.edu/ProteoSAFe/status.jsp?task=6ce593f186c045f985138a62548f43fd).

### MALDI mass spectrometry imaging (MSI)

To survey the spatial distribution of LPs production on the beads a MALDI-MSI experiment was performed on cryosections prepared after days of incubation following a modified method introduced by Kawamoto et al. [39]. The hydrogel beads were carefully picked from the microcosms and embedded in 2% (w/v) carboxy methyl cellulose (CMC, Thermo Fisher) solution in plastic molds (Polysciences, INC, USA). Immediately the samples were frozen at −80 °C and storage until cryosectioning. Embedded samples were cross-sectioned at −24 °C using a SLEE MEV cryostat (SLEE medical GmbH, Nieder-Olm, Germany). The samples were freed from the plastic mold and mounted on a cryostat disk using SLEE Cryo Glue (SLEE medical GmbH, Nieder-Olm, Germany) The sample was trimmed until the point of interest and later cryo-film (SECTION-LAB Co. Ltd., Yokohama, Japan) was adhered to the flat surface, and 12 μm sections of embedded beads were sliced. The pieces of cryo-film with alginate sections on them were adhered to MALDI IntelliSlides (Bruker, Massachusetts, USA) using 2 Way Glue pen (Kuretake Co., Ltd., Nara-Shi, Japan) on the edges of the tape with the beads side facing up. Then the slides were covered by spraying 0.5 ml of 2,5-dihydrobenzoic acid (DHB) (40 mg/ml in ACN/MeOH/H_2_O (70:25:5, v/v/v)) in a nitrogen atmosphere and dried overnight in a desiccator prior to IMS measurement. The samples were then subjected to timsTOF flex (Bruker Daltonik GmbH) mass spectrometer for MALDI MSI acquisition in positive MS scan mode with 50 µm raster width and a mass range of 100–2000 Da. Calibration was done using red phosphorus. The settings in the timsControl were as follow: Laser: imaging 50 µm, Power Boost 3.0%, scan range 46 µm in the XY interval, and laser power 70%; Tune: Funnel 1 RF 300 Vpp, Funnel 2 RF 300 Vpp, Multipole RF 300 Vpp, isCID 0 eV, Deflection Delta 70 V, MALDI plate offset 100 V, quadrupole ion energy 5 eV, quadrupole loss mass 100 *m*/*z*, collision energy 10 eV, focus pre TOF transfer time 75 µs, pre-pulse storage 8 µs. After data acquisition, the data were analyzed using SCiLS v2023a software. All data was stored in Metaspace [40] under https://metaspace2020.eu/project/lozano-andrade_2023.

### Root colonization assay

The transparent soil microcosm was assayed for supporting plant growth and root colonization by *B. subtilis* in the early stages of tomato seedlings development (*Solanum lycopersicum* L., Maja Buschtomato, Buzzy Seeds, NL). Seeds were surface sterilized in Eppendorf tubes by shaking in an orbital mixer for 10 min in 1.5 ml of 2% sodium hypochlorite. Afterward, seeds were washed five times in sterile MiliQ water alternating centrifugation and removal of liquid solution. Then, 10 seeds were germinated on 15% agar for 3 days. Subsequently, seedlings were soaked on a P5_B1_*gfp*_ bacterial solution (1 × 10^6^ CFU/ml) for 10 min and placed in LEGO brick boxes containing 50 g of beads [41]. The root colonization was tracked by confocal laser scanning microscopy imaging (CLSM) as described previously [42, 43] for 10 days. Colonized roots were washed twice with sterile ddH_2_O and placed onto microscope slides. Images were captured in a Leica TCS SP8 microscopy. Green-fluorescent reporter excitation was performed at 488 nm, while the emitted fluorescence was recorded at 520/23 nm. For generating multilayer images, Z-stack series with 1 μm steps were acquired and processed with the software Fiji [44].

## Results

### A transparent soil microcosm for studying *B. subtilis* and other bacterial species chemical ecology

**Fig. 1:**
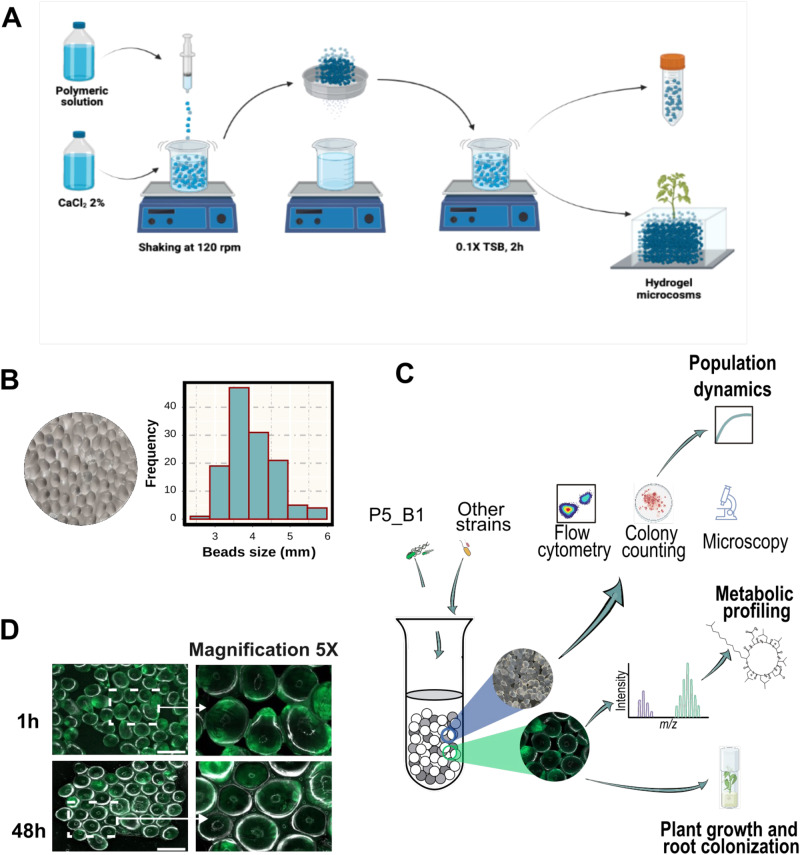
Overview of the hydrogel bead system for studying bacterial chemical ecology. **A** Hydrogel beads preparation protocol. A polymeric solution containing a mixture of Phytagel and sodium alginate is dropped into a solution of CaCl_2_. Then, the formed beads are soaked in 0.1 × TSB for 2 h. The excess of liquid is drained, and the beads are transferred into falcon tubes for subsequent experiments. **B** Hydrogel beads and size distribution. **C** Overview of the experimental approaches followed with the transparent soil microcosm and its possible applications. Beads within a given sample were mixed before downstream applications to reduce sampling heterogeneity. **D** Beads inoculated with P5_B1*gfp* inspected under fluorescence microscope.

In this study, we set up a transparent soil microcosm to investigate microbial interactions under axenic and soil-mimicking conditions. Our motivation for using this system came from the alginate bead-based method previously described by Ma et al. [13]. Following their protocol, we generated microcosms consisting of transparent beads ~3.97 ± 0.65 mm in diameter, enabling us to monitor bacterial population dynamics and metabolite production as indicators of bacterial establishment in the system. We aimed to assess bacterial growth and viability by coupling several techniques, including plate colony counting, flow cytometry, microscopy, and 16S rRNA gene profiling, with either pure cultures or co-cultivated strains within the microcosm. Furthermore, to detect and quantify lipopeptides produced by *B. subtilis*, we utilized UHPLC-HRMS and MALD-MSI, which allowed us to investigate bacterial interactions driven by this class of compounds within a controlled system. Thus, the system coupled to different analytic techniques provides a valuable tool for research on microbial community chemical ecology under defined laboratory conditions (Fig. 1).

### The transparent soil microcosm supports the growth of *B. subtilis* and other bacterial isolates

**Fig. 2:**
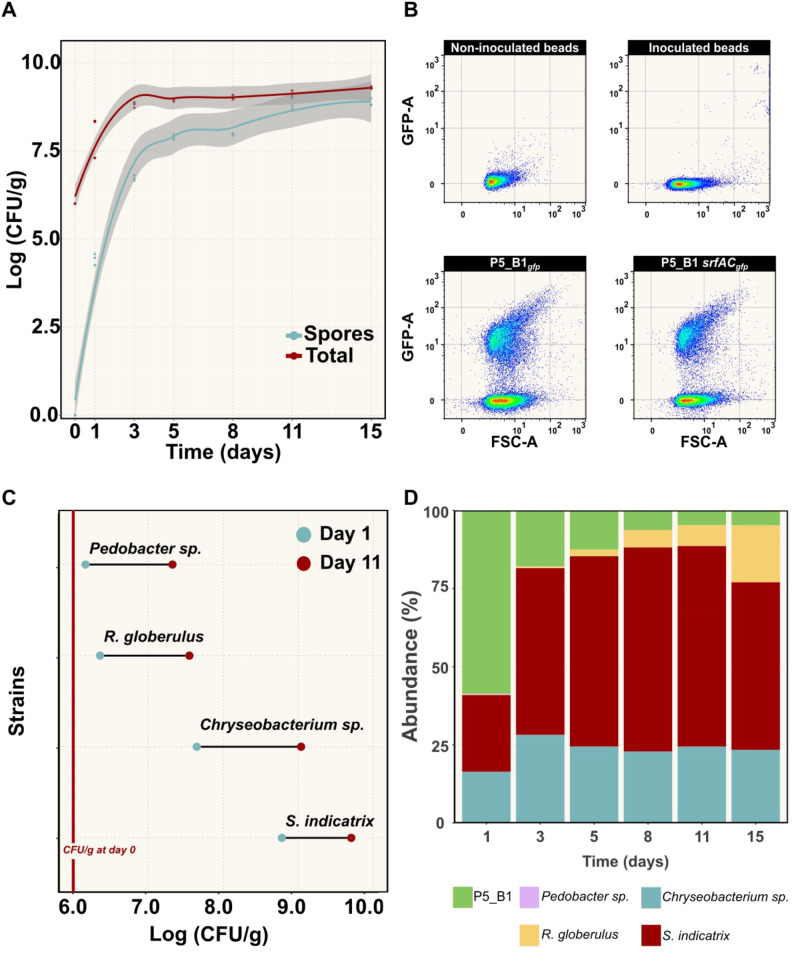
The transparent soil microcosm supports the growth of *B. subtilis* and other bacterial isolates. **A** Changes in *B. subtilis* P5_B1 populations (spore and total number of cells) on transparent soil microcosm were monitored as CFU/g over time (*n* = 3). The solid lines represent adjusted curve from a generalized model using the function stat_smooth in R. The gray area represents the dispersion given as confidence interval at 95%, and the points the actual count of each replicate. **B** Fluorescence intensity plot of *gfp*-labeled *B. subtilis* cells harvested from the beads at day 5 of inoculation. The gates were constructed from the non-fluorescent control samples. The GFP signal was detected using a 488 nm laser. **C** Endpoint population changes of four bacterial species on the soil microcosms. Population growth after day 1 and day 11 post inoculation were estimated by CFU/g (*n* = 3). **D** Culture-independent population dynamic estimation. A taxonomic summary showing the relative abundance of the five bacterial species inoculated into the transparent soil over 15 days (*n* = 3).

To prove that the transparent soil microcosm can support bacterial growth and development, we monitored the population dynamics of P5_B51_*gfp*_ for 15 days using plate count and flow cytometry (Fig. 2). According to the colony counting results, both the total number of cells and spores increased exponentially until day 5, followed by a plateau until the last sampling point (15 days post inoculation), with a maximum carrying capacity of ~1 × 10^9^ CFU/g. Sporulation is a critical phenotypic trait in *Bacillus* ecology since dormant spores may affect fitness and secondary metabolite production. In our single-specie microcosm experiments, the spore population varied from around 55%, at day 2, to a maximum level of around 88% after 15 days (Fig. 2A).

In addition, to increase the throughput on tracking *B. subtilis* populations on the hydrogel beads, we explored the use of flow cytometry for cell counting, taking advantage of our strain’s constitutive expression of green fluorescent protein (GFP) (Fig. 2C). We set up proper control groups, including non-inoculated beads, beads inoculated with P5_B1WT, and medium (0.1 × TSB), and quantified GFP-positive cells using FlowJo v10.8.2. Our flow cytometry-based approach revealed that P5_B1 followed an exponential growth on the beads up to day 5, increasing by approximately two orders of magnitude before reaching a plateau by day 8 onwards, in line with results obtained through plate counting (Fig. S1).

To further investigate the system’s ability to support bacterial growth, we evaluated whether other strains, isolated from the same soil sample site as *B. subtilis* P5_B1, could grow on the hydrogel matrix supplemented with 0.1 × TSB. Colony counting revealed that the transparent soil microcosm could sustain the growth of all four soil-derived bacterial isolates. Assessment of the growth properties confirmed that all strains were able to grow and increase their population by at least two orders of magnitude compared to the initial inoculum size (~10^6^ CFU/g) at day 11 post inoculation (Fig. 2C).

Additionally, we surveyed whether the microcosms are suitable to grow a bacterial assemblage and estimate its composition using a culture-independent method that relies on environmental DNA extractions. As expected, we obtained high-quality and quantity total DNA (>220 ng/µl) from the system due to the lower complexity of the transparent soil compared to soil. Using 16S rRNA gene sequencing, we monitored the bacterial community composition over a 15-day period. At day 1, we detected all the five strains at different proportions, being P5_B1*gfp* the most abundant strain because of its initial inoculation ratio (1:20). Over time, the bacterial assemblage composition changed, while *S. indicatrix* and *R. globerulus* increased their population, P5_B1*gfp* decreased to around 10% of its abundance, while *Pedobacter* sp. was below detection limit. By the end of the experiment, *S. indicatrix* and *Chryseobacterium* sp. were the dominant taxa of the bacterial community (Fig. 2D).

### Surfactin and plipastatin can be detected in the transparent soil microcosms

**Fig. 3:**
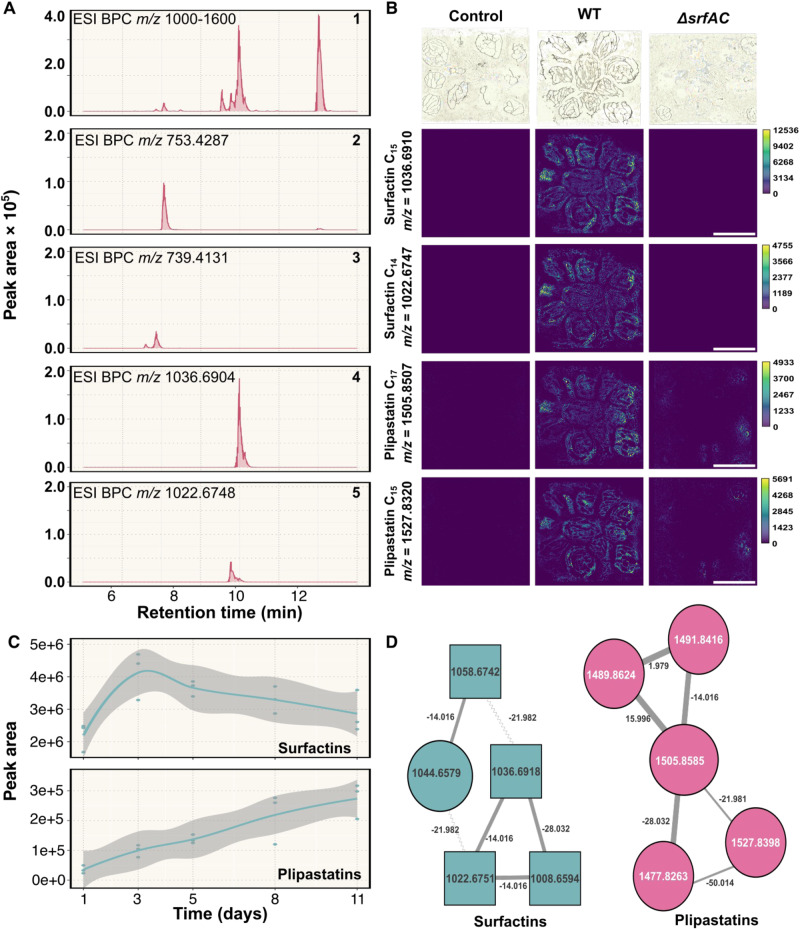
Surfactin and plipastatin are produced at detectable levels in the transparent soil microcosms. **A** Base peak chromatograms (BPC) of *B. subtilis* lipopeptides detected on the hydrogel matrix at day 5: (1) lipopeptides detection (*m/z* 1000–1600), (2) Plipastatin B C_17_ (*m/z* 753.4287 ± 10 ppm), (3) Plipastatin A C_17_ (*m/z* 739.4131 ± 10 ppm), (4) Surfactin-C_15_ (*m/z* 1036.6904 ± 10 ppm), (5) Surfactin-C_14_ (*m/z* 1022.6748 ± 10 ppm). Beads within a given sample were mixed before extraction to reduce sampling heterogeneity. **B** MSI spectrometry reveals the presence/absence of isoforms of the surfactin and plipastatin families in each *B. subtilis* variant. Scale bar indicates 5 mm. **C** Surfactin and plipastatin dynamics over the time measure as the peak area of each compound. **D** Refined molecular network of the metabolic profile of P5_B1 propagated on the beads. Molecular network after filtering the complete network with parent masses between 700 and 1600, RTmean from 6 to 11 min.

One bottleneck in chemical ecology research on *Bacilli* is imposed by the difficulty to detect and quantify LPs and other secondary metabolites in their niche where these are naturally produced, limiting our understanding of how those compounds impact the ecology of the producing organisms and other interacting species. Therefore, to enlighten the qualitative productions of LPs in our experimental system, we monitored the metabolic profile of P5_B1 using UHPLC-HRMS, targeting compounds with *m/z* values between 700 and 1600 (to include doubly charged plipastatins), which is the typical *m/z* range for *Bacillus* LPs detection [45]. Using a combination of GNPS molecular networking and targeted dereplication (NPAtlas) [33, 46], we detected multiple isoforms belonging to surfactin and plipastatin families in the axenic cultures of P5_B1, with surfactin C_14_ and plipastatin B C_17_ being the most abundant features of the LP mixture (Fig. 3A, D and Table 2). In these experimental conditions, we observed a dynamic production of surfactin and plipastatin over the time. Surfactin reached its maximum (peak area) at day 3, which coincided with the late exponential phase of bacterial growth in the system. On the other hand, the peak of plipastatin production was delayed until day 8, when the bacterial growth plateaued (Fig. 3C). Both observations fall in line with what is typical observed when analyzing liquid fermentations.

**Table 2:** Identified lipopeptides.

Retention time (min)	*m/z*	ID	Formula
10.73	753.4292 [M + 2H]^2+^, 1505.8507 [M + H]^+^	Plipastatin B C_17_	C_75_H_116_N_12_O_20_
10.08	739.4141 [M + 2H]^2+^	Plipastatin B C_15_	C_73_H_112_N_12_O_20_
10.45	739.4127 [M + 2H]^2+^	Plipastatin A C_17_	C_73_H_112_N_12_O_20_
10.62	731.4169 [M + 2H]^2+^	Plipastatin B C_14_	C_73_H_112_N_12_O_19_
11.02	731.4148 [M + 2H]^2+^	Plipastatin A C_16_	C_73_H_112_N_12_O_19_
11.24	745.4305 [M + 2H]^2+^, 1489.8552 [M + H]^+^	Plipastatin B C_16_	C_75_H_116_N_12_O_19_
13.35	1008.6593 [M + H]^+^, 1030.6409 [M+Na]^+^	Surfactin-C_13_^a^	C_51_H_89_N_7_O_13_
13.66	1022.6764 [M + H]^+^, 1044.6580 [M+Na]^+^	Surfactin-C_14_^a^	C_52_H_91_N_7_O_13_
13.97	1036.6910 [M + H]^+^, 1058.6728 [M+Na]^+^	Surfactin-C_15_^a^	C_53_H_93_N_7_O_13_

^a^Validated by LCMS of authentic standard.

To corroborate the HPLC-MS findings and dissect the spatial distribution of surfactin and plipastatin in our experimental system, we tracked LP production by MALDI MS imaging. The isoforms from surfactins (C_14_ and C_15_) and plipastatins C (C_15_ and C_17_) were detected in all bead section surveyed, confirming that those metabolites are diffusible in the matrix as well as detectable on the surface of the alginate beads. As expected, beads inoculated with a *srfAC* mutant lacked surfactin production (Fig. 3). Importantly, the alginate beads have their own molecular signature (*m*/*z* 657.144), therefore allowing us to distinguish their background from metabolites produced by the bacteria (Fig. S2).

### The transparent soil microcosm allows plant growth and serves as gnotobiotic system for studying *B. subtilis* root colonization

**Fig. 4:**
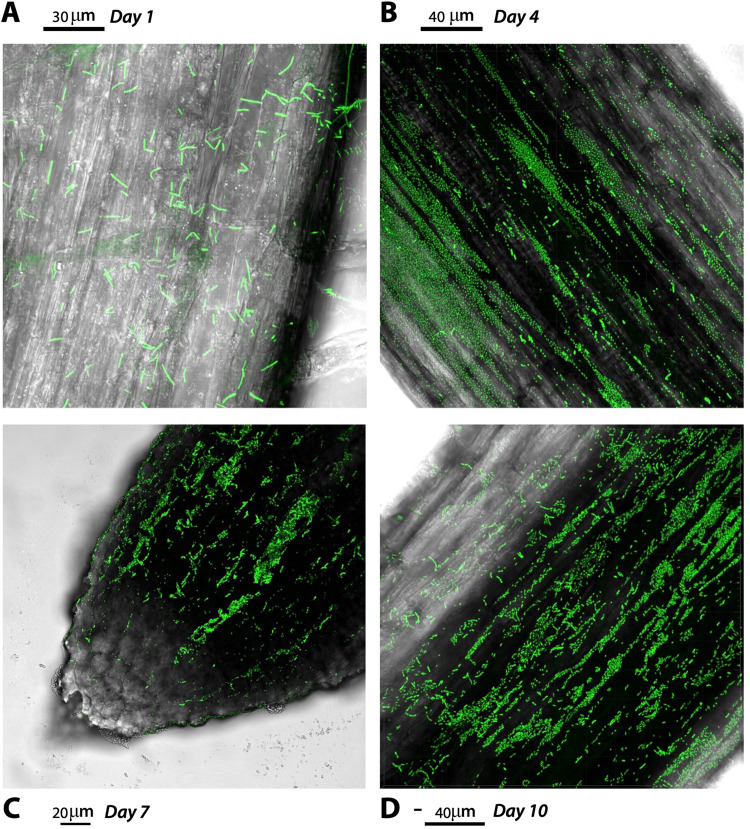
Tomato roots colonized by *B. subtilis* P5_B1*gfp*. The panel shows the P5_B1 colonization dynamics over 10 days (**A** to **D**: 1, 4, 7, and 10 days, respectively). The tomato seedlings were grown on the hydrogel beads and imaged by CLSM. Images are representative of three independent tomato seedlings and showed actively colonized root tips. The scale bars are indicated in each image.

To examine whether the microcosm can support plant growth and serve as model for *B. subtilis* root colonization assays on gnotobiotic conditions, pre-germinated tomato seeds were inoculated with a bacterial suspension and placed on the plant cultivation box based on LEGO assemblies [41]. Overall, the plants emerged after 4 days post inoculation and, during 2 weeks of growth, the leaves appeared healthy and dark green. Notably, the roots system grew profusely allowing subsequent inspection of P5_B1_*gfp*_ colonization under CLSM. Here, P5_B1_*gfp*_ formed robust biofilm on the root and fluorescent cells were detected up to 10 days post inoculation, suggesting that the system can be used for interrogating the role of LPs in root colonization in early stages (Fig. 4).

## Discussion

Secondary or “specialized” metabolites are a major driver of interactions among species in the microbial world [47, 48]. Recently, understanding why and how microbes produce and use these remarkable molecules in their natural habitat has become a research focal point [6, 8, 49]. As such, natural products may shape the composition and activity of microbiomes through multiples mechanisms varying from signaling to niche defense [50]. However, the role of SMs under natural conditions are scarcely revealed and their study is constrained by limitations in detecting and quantifying SMs in environmental matrices [7, 51].

To overpass this limitation and evaluate the ecological roles of these specialized natural product, model systems of intermediate microbial and chemical diversity are required [52]. Here, we explored the use of a transparent hydrogel matrix developed by Ma et al. as a soil-like model system for studying *B. subtilis* chemical ecology combining microbial and chemical methods.

While transparent soils and soil-like microcosms have been used to investigate plant-microbe interactions, to our knowledge, none of them have been proposed or utilized as controllable systems to interrogate secondary metabolite production and bacterial population dynamics. In this work, we demonstrated how lipopeptide production by *B. subtilis* can be tracked both temporally and spatially by coupling HPLC-MS and MALDI MS imaging, overcoming limitations associated with extraction, detection, and quantification of this class of compounds in soil. When propagating P5_B1 on hydrogel beads supplemented with the diluted complex medium, 0.1 × TSB, we found that surfactin production was higher than the plipastatin production in all the sampling times. However, the two metabolites production followed different temporal dynamics. Specifically, surfactin reached its peak at day 3, concurring with the late exponential phase of bacterial growth in the system, while plipastatin peak was delayed until day 8 when the bacterial growth plateaued. These observations align with earlier studies conducted on planktonic cultures where surfactin is produced toward the end of the early exponential growth phase, while other LPs are produced later during the stationary growth phase [53, 54]. Interestingly, we observed that the strains produced a mixture of isoforms from surfactin and plipastatin families at detectable levels in the hydrogel matrix. This phenomenon has been widely described for several *Bacillus* strains growing under various conditions [55–59]. However, given the challenges posed by natural soil, the relevance of producing such a diversity of compounds with minor structural changes has been poorly explored. Therefore, our hydrogel matrix can be considered as a potential system for testing how the composition of the isoform mixture could affect the behavior of the producer (e.g., biocontrol activity, motility, spreading, or survival) and its interactions with other microbes in a structured environment.

As Ma et al. demonstrated, the hydrogel transparent soil produces field-relevant root phenotypes in *Glycine max* [13]. Therefore, we also interrogated whether bacterial-inoculated tomato seedlings could be grown on the system and studied root colonization pattern by *B. subtilis*. As we expected, the microcosms provided optimal controlled growth conditions for tomato and the plant-associated bacterium *B. subtilis* facilitating the study of host-microbe interactions possibly influenced by secondary metabolites in the rhizosphere.

Overall, the described system provides numerous experimental advantages and allows for a high degree of customization. However, it is important to acknowledge that, like any lab-scale simplified experiment, there are inherent limitations compared to studies conducted in natural soil or other soil-like systems. Firstly, the hydrogel matrix is less complex than natural soil in terms of organic matters composition. As described earlier, the primary carbon sources in our system come from the polymeric matrix (sodium alginate and Phytagel) and TSB medium. However, these sources may not accurately reflect the complex mixture of organic compounds intricately associated with the mineral matrix found in natural soils [60, 61]. Similarly, as Ma et al. demonstrated that the effective porosity reached with beads around 5 mm in diameter is comparable to sandstone [13]. These two factors, the low complexity of organic matters and structure in the transparent soil microcosm, may alter microbial interactions and community assembly patterns since they play a significant role in shaping microbial dynamics in their natural habitats [62–64]. Additionally, the use of hydrogel beads as a matrix presents limitations for single-cell level microbial visualization due to their size (~400 µm), which may impede detailed microscopic analysis like the one explored by Sharma et al. using Nafion and cryolite particles mounted in a microfluidic chamber [12]. Furthermore, phytagel and sodium alginate, the major components of the beads, may be susceptible to degradation by certain microbial species that can utilize them as a carbon source [65, 66].

In summary, this study provides a proof-of-concept for the use of a transparent hydrogel matrix as a soil-like model system to investigate the production and ecological roles of specialized metabolites by *B. subtilis*. By combining microbial and chemical methods, we were able to track the temporal and spatial dynamics of lipopeptide production by *B. subtilis* on the hydrogel beads. Moreover, we demonstrated the applicability of the system for studying plant-microbe interactions by exploring a key trait, the ability of *B. subtilis* to actively colonize plant roots [43, 67, 68].

Notably, the described experimental system can facilitate the dissection of the importance of richness (number of interacting species) and structure (how the members contribute to the overall community performance) on bacterial interaction where SM production may be relevant. Furthermore, in the future, the transparent hydrogel matrix could be used in combination with other techniques that are designed to simplify soil microbiology studies, such as standardized soil growth media and simplified soil microbial communities to mechanistically study community-level interactions driven by bacterial secondary metabolism.

### Supplementary information

Figure S1 and S2

## Data Availability

The amplicon sequencing data are available in NCBI SRA: PRJNA982715. The FBMN workflow is available in GNPS: https://gnps.ucsd.edu/ProteoSAFe/status.jsp?task=6ce593f186c045f985138a62548f43fd. The MALDI-MSI data are available in Metaspace: https://metaspace2020.eu/project/lozano-andrade_2023.
