# Supplementary material for: Establishment of a transparent soil system to study Bacillus subtilis chemical ecology
Source: ISME Commun. 2023 Oct 14;3:110. doi: 10.1038/s43705-023-00318-5 (PMC10576751; doi:10.1038/s43705-023-00318-5)
Supplement: Supplementary file 1 — Figure S1 and S2 [file 43705_2023_318_MOESM1_ESM.pdf]

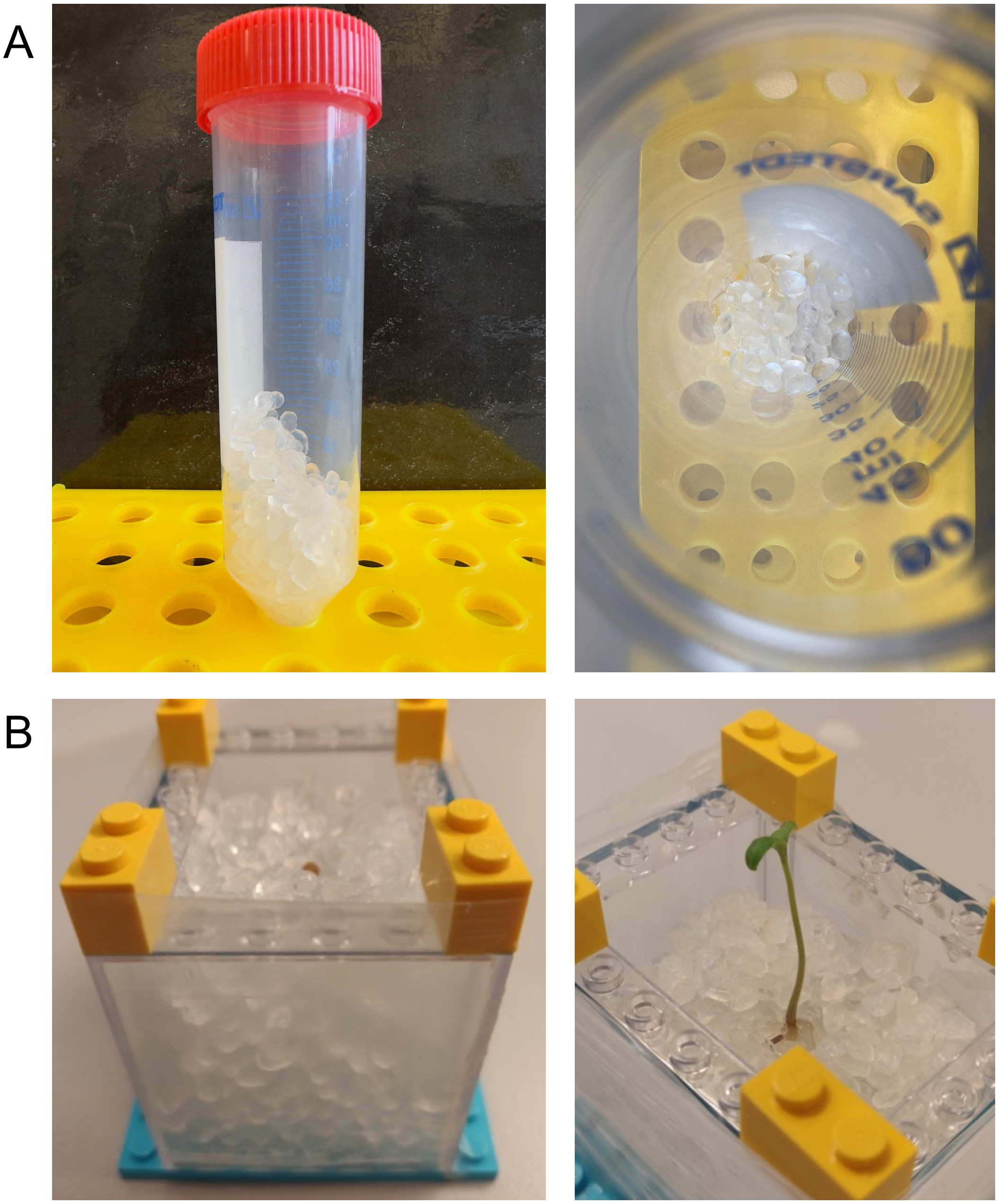

Fig S1 Hydrogel beads soil microcosmos. A) Falcon tube microcosm. 25 mL of freshly prepared beads were poured into sterile 50 mL Falcon tubes. This setup was used for all the experiments where *B. subtilis* or the other bacterial strains were evaluated in their ability to grow and produce secondary metabolites in the soil-like matrix. B) The LEGO brick box microcosm. The LEGO boxes were used for the plant root colonization assays exclusively.

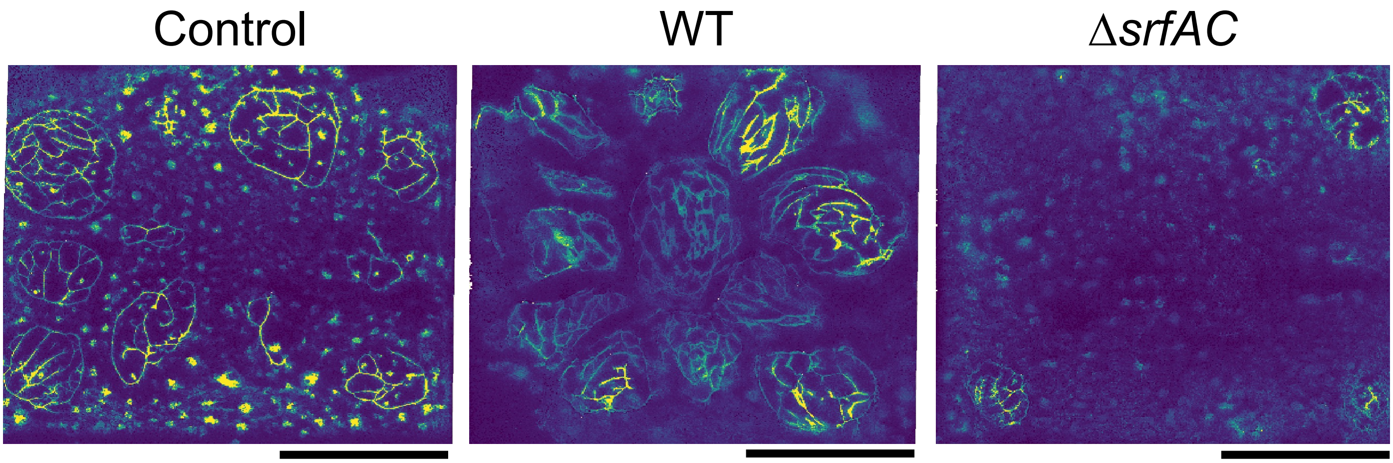

Fig S2 MSI spectrometry targeting a unique feature from the hydrogel matrix (*m/z* 657.144) allowing to distinguish the beads shapes from the metabolites produced by *B. subtilis* P5_B1. Scale bars indicate 5mm.
